# Hyperglycaemia Exacerbates Choroidal Neovascularisation in Mice via the Oxidative Stress-Induced Activation of STAT3 Signalling in RPE Cells

**DOI:** 10.1371/journal.pone.0047600

**Published:** 2012-10-19

**Authors:** Xia Li, Yan Cai, Yu-Sheng Wang, Yuan-Yuan Shi, Wei Hou, Chun-Sheng Xu, Hai-Yan Wang, Zi Ye, Li-Bo Yao, Jian Zhang

**Affiliations:** 1 Department of Ophthalmology, Xijing Hospital, Fourth Military Medical University, Xi’an, Shaanxi Province, People’s Republic of China; 2 State Key Laboratory of Cancer Biology, Department of Biochemistry and Molecular Biology, Fourth Military Medical University, Xi’an, Shaanxi Province, People’s Republic of China; 3 Department of Orthopedics, Xijing Hospital, Fourth Military Medical University, Xi’an, Shaanxi Province, People’s Republic of China; 4 State Key Laboratory of Cancer Biology, Department of Gastrointestinal Surgery, Xijing Hospital, Fourth Military Medical University, Xi’an, Shaanxi Province, People’s Republic of China; University of Florida, United States of America

## Abstract

Choroidal neovascularisation (CNV) that occurs as a result of age-related macular degeneration (AMD) causes severe vision loss among elderly patients. The relationship between diabetes and CNV remains controversial. However, oxidative stress plays a critical role in the pathogenesis of both AMD and diabetes. In the present study, we investigated the influence of diabetes on experimentally induced CNV and on the underlying molecular mechanisms of CNV. CNV was induced via photocoagulation in the ocular fundi of mice with streptozotocin-induced diabetes. The effect of diabetes on the severity of CNV was measured. An immunofluorescence technique was used to determine the levels of oxidative DNA damage by anti-8-hydroxy-2-deoxyguanosine (8-OHdG) antibody, the protein expression of phosphorylated signal transducer and activator of transcription 3 (p-STAT3) and vascular endothelial growth factor (VEGF), in mice with CNV. The production of reactive oxygen species (ROS) in retinal pigment epithelial (RPE) cells that had been cultured under high glucose was quantitated using the 2′,7′-dichlorofluorescein diacetate (DCFH-DA) method. p-STAT3 expression was examined using Western blot analysis. RT-PCR and ELISA processes were used to detect VEGF expression. Hyperglycaemia exacerbated the development of CNV in mice. Oxidative stress levels and the expression of p-STAT3 and VEGF were highly elevated both in mice and in cultured RPE cells. Treatment with the antioxidant compound N-acetyl-cysteine (NAC) rescued the severity of CNV in diabetic mice. NAC also inhibited the overexpression of p-STAT3 and VEGF in CNV and in RPE cells. The JAK-2/STAT3 pathway inhibitor AG490 blocked VEGF expression but had no effect on the production of ROS *in vitro*. These results suggest that hyperglycaemia promotes the development of CNV by inducing oxidative stress, which in turn activates STAT3 signalling in RPE cells. Antioxidant supplementation helped attenuate the development of CNV. Thus, our results reveal a potential strategy for the treatment and prevention of diseases involving CNV.

## Introduction

Age-related macular degeneration (AMD) is a major cause of visual impairment in elderly people. Choroidal neovascularisation (CNV) beneath the macula, which occurs in the late stage of the disease and is characterised as the “wet form” of AMD, causes rapid central vision loss that has serious effects on the quality of life in older patients [1]. A number of genetic and environmental factors have been identified as being risk factors for neovascular AMD, and knowledge of these factors has helped in both preventing and reducing the occurrence and process of the disease [2].

Because of the effect that diabetes has on vascular systems, epidemiological studies have focused on the relationship between diabetes and AMD. The similarity of the results that were obtained from an ancillary study to the Women’s Health Initiative Sight Exam and a clinical study concerning the association between myocardial infarctions and the development of AMD suggested that diabetes is a risk factor for AMD [3], [4]. However, other studies have come to contrary conclusions [5], [6], [7], [8]. In fact, even studies that investigated risk factors that were associated with different types of AMD had inconsistent conclusions regarding whether diabetes was a factor that affected the disease [5], [9]. Moreover, two abstracts that were presented at the annual meeting of the Association for Research in Vision and Ophthalmology (ARVO) found that diabetes enhanced the development of laser-induced CNV in mice but suggested that understanding the underlying mechanism for this enhancement required further investigation [10], [11]. Therefore, in the present study, we were interested in not only identifying the association between diabetes and CNV but also in attempting to investigate the underlying mechanisms that were responsible for the association.

Increasing evidence has shown that oxidative stress contributes to the development of a wide range of diseases, including age-related diseases, cancer, metabolic diseases and neural diseases. Although the pathogenesis of CNV remains uncertain, it has been suggested that oxidative stress plays a causative role in both the initiation and progression of CNV [12]. It has been reported that mice that are deficient in Cu, Zn-superoxide dismutase (SOD1) have features that are typical of AMD in humans such as the presence of drusen, thickening of Bruch’s membrane and CNV [13]. It has been confirmed that the downregulation of NADPH oxidase-mediated ROS production in the retinal pigment epithelial (RPE) cells of mice reduces CNV lesions [14]. In addition, studies have demonstrated that the use of antioxidant supplementation to counter cellular oxidative stress results in the suppression of experimental CNV [15]. Meanwhile, diabetes-related investigations have shown that oxidative stress is a key factor in the initiation of structural and functional vascular changes [16], [17]. It has been reported that generation of ROS is responsible for early stages of diabetic nephropathy [18]. Other studies have demonstrated that hyperglycaemia results in an increase in the production of superoxide in retina and ultimately contributes to the pathogenesis of diabetic retinopathy (DR) [19]. Furthermore, treatments that reduce the formation of ROS were successful in preventing DR in a streptozotocin (STZ)-induced diabetic rat model [20].

The signal transducer and activator of transcription-3 (STAT3) protein is important for the regulation of cell differentiation, proliferation, and angiogenesis [21]. Previous studies have verified that STAT3 is a direct transcriptional activator of the vascular endothelial growth factor (VEGF) gene [22]. In a murine model of laser-induced CNV, STAT3 activation was found to be involved in promoting the development of CNV [23]. Recent findings have suggested that diabetes increases the level of STAT3 activation and thereby contributes to the pathophysiology of vascular injury [20].

In the present study, we have investigated the effects of diabetes on the development of laser-induced CNV in mice, and we have also investigated the roles that oxidative stress and STAT3 signalling play in the regulation of VEGF in RPE cells in a high glucose environment.

## Results

### Blood Glucose and Body Weight after Injection of Streptozotocin

**Figure 1:**
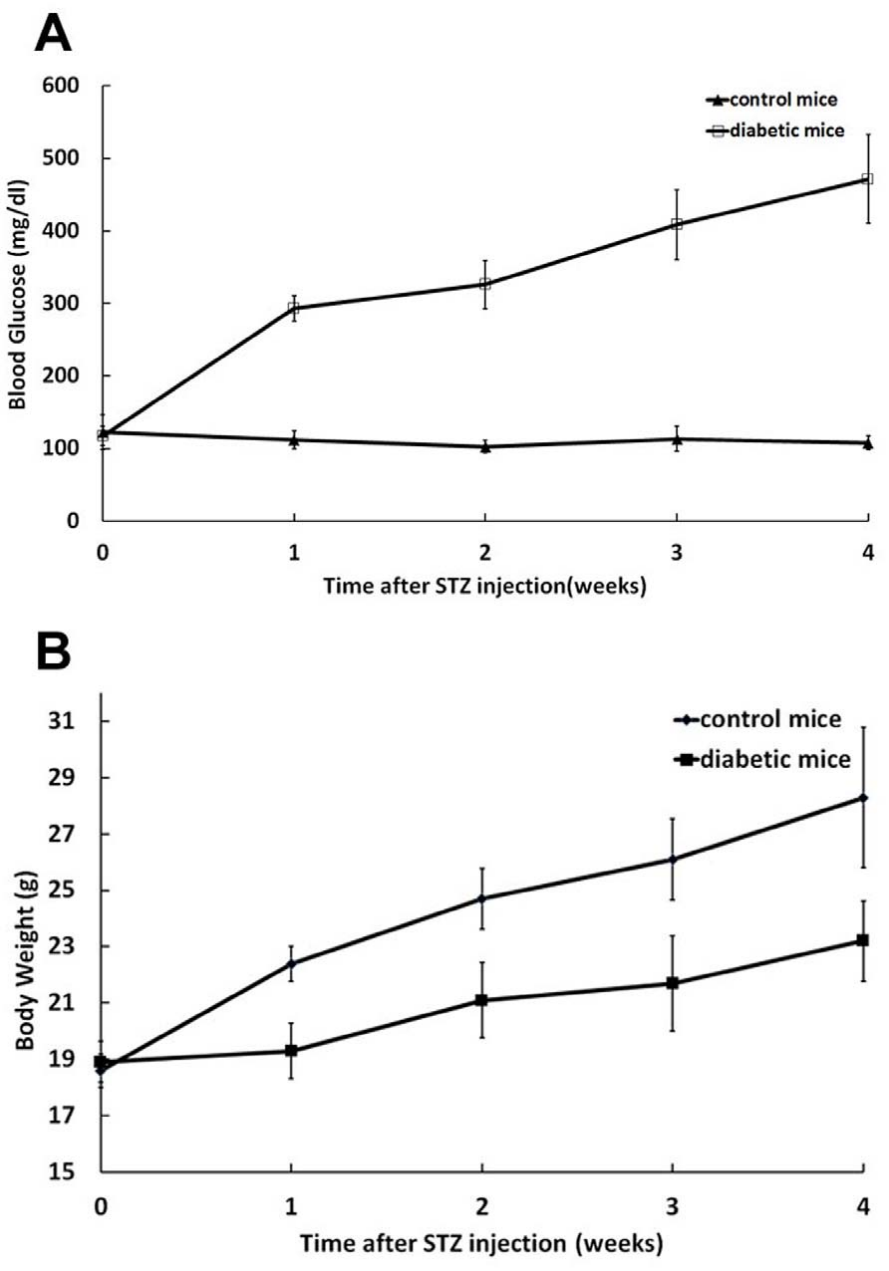
Effects of streptozotocin on blood glucose and body weight of mice. A, STZ injection resulted in elevated blood glucose levels in mice. B, Diabetic mice had lower body weight gain in compared to control group. Error bars represent ± SEM. All diabetic time points are significantly different from control (*P*<0.05).

Streptozotocin injection significantly elevated blood glucose levels compared to control mice at 5 time points throughout the experimental period: pretreatment, 1-week, 2-weeks, 3-weeks and 4-weeks post-STZ injection (Fig. 1 A). Body weights of animals were assessed starting on the day of injection and followed thereafter at one-week intervals to observe changes in weights. Control mice showed an increase in body weight over the experimental period, but the diabetic mice had lower weight gain compared with control group (Fig. 1 B).

### Hyperglycaemia Promoted the Formation of CNV in Mice

**Figure 2:**
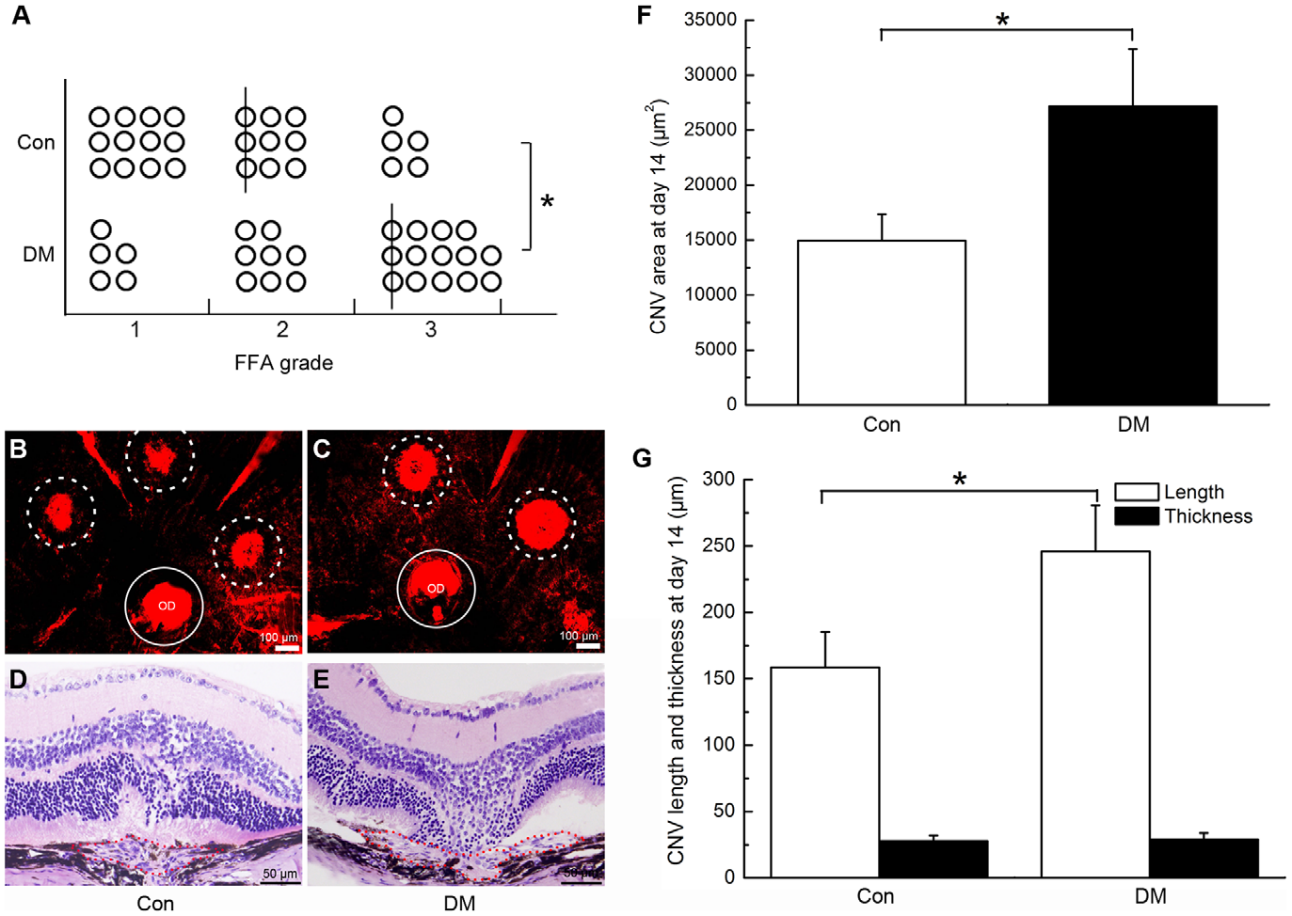
Hyperglycaemia promoted the development of CNV 2 weeks after laser photocoagulation. A, Statistical analysis of the fluorescein leakage in control mice (Con) and diabetic mice (DM) (*P<0.01, lines indicate median leakage levels). B–C, Representative flatmount preparations of the eyecups of control (B) and diabetic (C) mice (Dotted circles: area of CNV; OD: optic disc; Circles: area of OD). D–E, H&E staining images of serial cross-sections of the eye cups of control (D) and diabetic (E) mice. F, Statistical analysis of the data in B and C (*P<0.01); G, Statistical analysis of the data in D and E (*P<0.01).

Vascular complexes that formed after the damage to Bruch’s membrane extended from the choroid to the subretinal space and caused an appearance of hyperfluorescence in FFA. The leakage reflects the permeability of the neovascularization. Although the difference between the incidences of CNV at the irradiated spots in the diabetic and control mice was not significant (*P*>0.05), the degree of fluorescence leakage in the eyes of the hyperglycaemic mice was significantly elevated relative to that in the control mice on day 14 after photocoagulation (*P<0.01, Fig. 2 A).

Similar results were found in both the flatmount and histopathology assays. Compared with mice in the control group, the average area of CNV was markedly larger in diabetic mice on day 14 (14931±2432 vs. 27162±5197 µm^2^ for control and diabetic mice, respectively; *P<0.01; Fig. 2 B, C and F). However, analysis of the cross-sectional slices revealed that there was no significant difference between the average thicknesses of CNV in the two groups (27.8±4.2 vs. 29.1±4.6 µm for control and diabetic mice, respectively; *P*>0.05; Fig. 2 D, E and G) and that the CNVs in hyperglycaemic mice were wider than those of the control group (158.6±26.7 vs. 245.9±34.7 µm for control and diabetic mice, respective; *P<0.01; Fig. 2 D, E and G).In general, we found that the width and surface area of the CNV lesions had a significant difference but the thicknesses had no difference between the two groups. One possible explanation is that after photocoagulation-induced rupture of Bruch’s membrane in mice, vascular complexes began to grow and extend from the choroid to the subretinal space. Because of the relatively tight junctions of the inner retina that possessed, the progression of CNV was mainly located at the external retinal layer. This characteristic causes pathological neovascularization grow parallel to the plane of retinal layer but hard to develop upwards vertically [24]. We thought that this may be the reason that help to explain why there was no significant difference in the thickness of the CNV lesions in the two groups.

### Hyperglycaemia Increased Oxidative DNA Damage, Up-regulated VEGF and Phosphorylated STAT3 (p-STAT3) Expression in CNV

**Figure 3:**
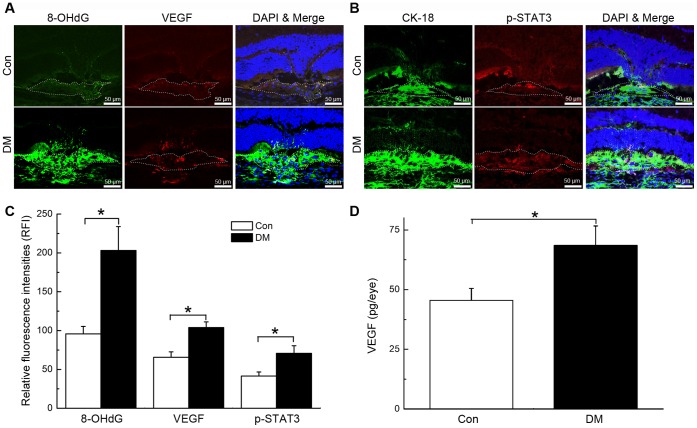
Hyperglycaemia resulted in the up-regulation of 8-OHdG, VEGF and p-STAT3 expression at sites of CNV. A-B, 8-OHdG, VEGF and p-STAT3 expression patterns at CNV sites in control (Con) and diabetic (DM) mice. CNV lesions were encircled with dashed lines. Relative fluorescence intensities (RFI) of targeted proteins were measured by analysing images of the immunofluorescence staining of serial cross sections of the eyecups. C, Statistical analysis of the data in A and B (*P<0.01); D, Statistical analysis of the data from the VEGF ELISA (*P<0.01).

Local expression patterns of 8-OHdG, VEGF and p-STAT3 in the initial stage of experimentally induced CNV formation were investigated to determine a potential underlying mechanism for the effects of hyperglycaemia. Compared with the control group, we found evidence of elevated levels of oxidative DNA damage (95.8±9.6 vs. 203.2±30.7 RFI in control and diabetic mice, respectively; *P<0.01; Fig. 3 A and C), upregulateion of VEGF (65.7±6.9 vs. 103.9±7.3 RFI in control and diabetic mice, respectively; *P<0.01; Fig. 3 A and C) and p-STAT3 (41.5±5.2 vs. 70.8±9.8 RFI in control and diabetic mice, respectively; *P<0.01; Fig. 3 B and C) expression in the choroid beneath CNV lesions in the eyes of diabetic mice on day 3 after laser damage. An ELISA further confirmed that the upregulation of VEGF expression was induced by hyperglycaemia (45.5±5.0 vs. 68.5±8.1 pg/eye in control and diabetic mice, respectively; *P<0.01; Fig. 3 D).

### High Levels of Glucose Promoted Intracellular ROS Formation, STAT3 Activation and VEGF Production in RPE Cells

**Figure 4:**
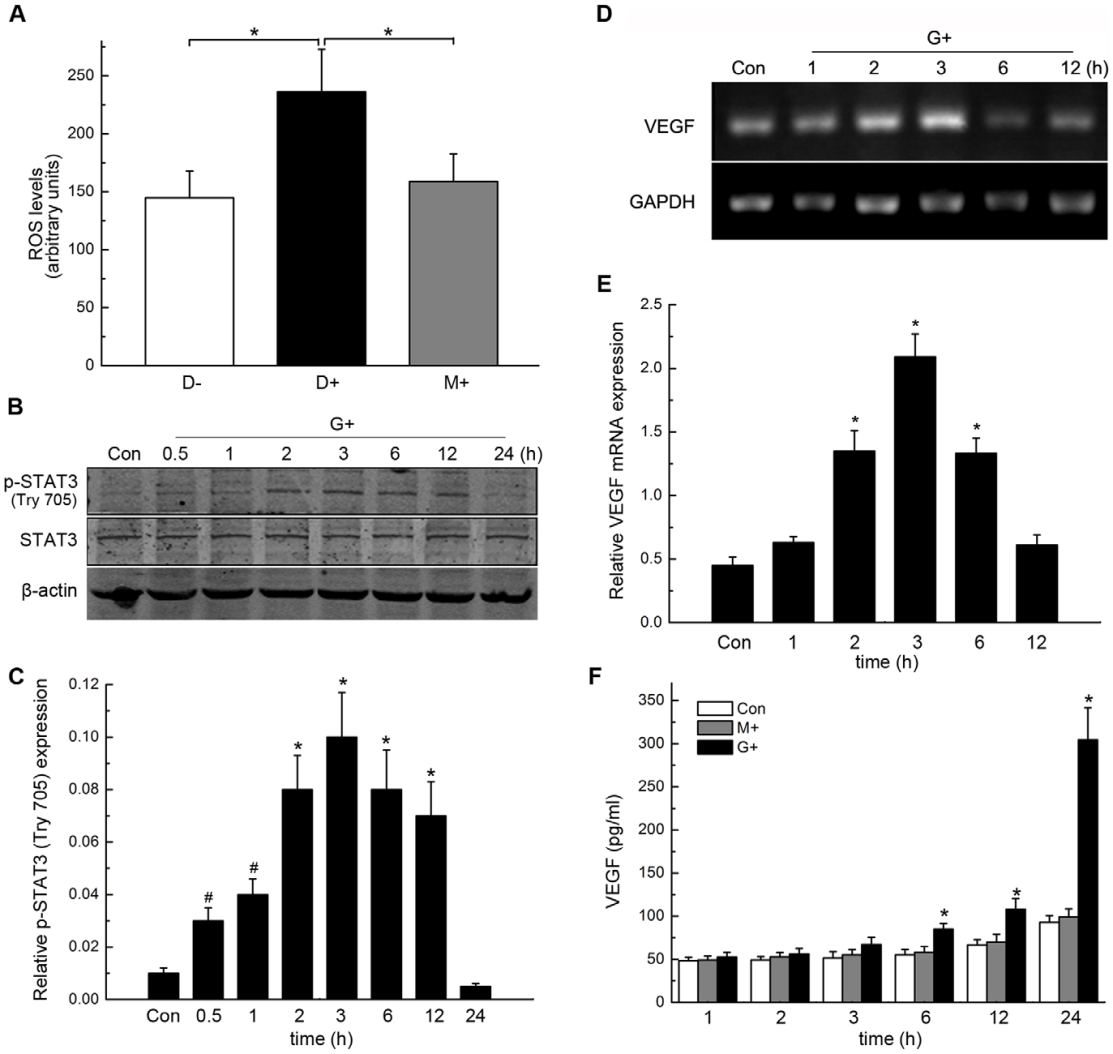
High glucose (HG) promoted intracellular ROS formation, STAT3 activation and VEGF production in RPE cells. A, compared with that were treated with high mannitol (HM) and normal glucose (NG) solutions, treatment with a HG solution increased the ROS level in RPE cells (*P<0.01). B, expression of p-STAT3 and STAT3 in RPE cells after different lengths of exposure to an HG solution as measured by Western Blotting. The results from 3 representative experiments that were performed independently are shown. C, Statistical analysis of the data in B (*P<0.01, #P<0.05). D, Results of representative RT-PCR experiments showing the levels of VEGF mRNA expression at the indicated times. E, Statistical analysis of the data in D (*P<0.01). F, Statistical analysis of the VEGF ELISA data (*P<0.01).

Because the retinal pigment epithelium has a higher rate of oxygen consumption than any other tissue, RPE cells are vulnerable to oxidative damage [25]. To confirm the role of hyperglycaemia and its subsequent effects on the development of CNV, we investigated the levels of intracellular ROS formation, STAT3 activation and VEGF production in RPE cells. The levels of ROS that were found in the high glucose group are significantly higher than those of the low glucose and mannitol groups (*P<0.01, Fig. 4 A).

The level of p-STAT3 protein expression in RPE cells significantly increased in a time-dependent manner; it reached a maximum after 3 hours of exposure to a high-glucose medium and subsequently decreased (*P<0.01, #*P*<0.05, Fig.4 B–C), whereas the total level of STAT3 protein expression changed weakly.

In addition, the amount of VEGF mRNA in RPE cells was also up-regulated during exposure to a high glucose medium, and the timecourse of the up-regulation was similar to that of the change in p-STAT3 protein expression (*P<0.01, Fig. 4 D–F). After 6 h, the amount of VEGF protein that had been secreted by RPE cells in a culture medium that contained a high concentration of glucose was also higher than the amount of VEGF that had been secreted by RPE cells in a control medium (*P<0.01, Fig. 4 F).

### NAC-induced Suppression of Oxidative Stress Rescued CNV Severity

**Figure 5:**
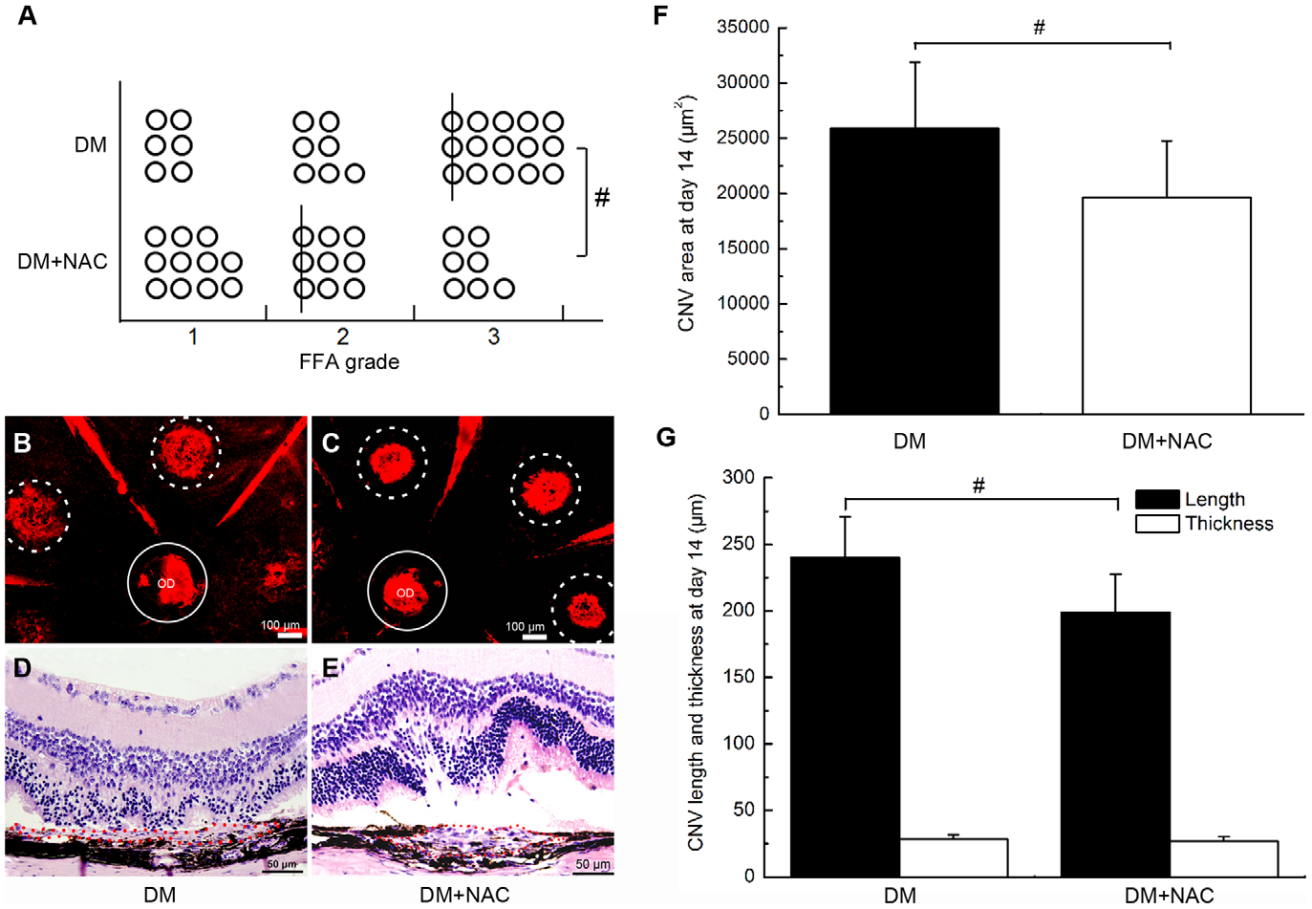
NAC-induced suppression of oxidative stress rescued CNV severity. A, Statistical analysis of the fluorescein leakage in untreated diabetic (DM) and NAC-treated diabetic (NAC) mice (#P<0.05); lines indicate the median CNV grades. B-C, Areas of CNV lesions in the aforementioned groups; D–E, H&E staining of CNV lesions in DM and NAC mice; F, Statistical analysis of the data presented in B and C (#P<0.05). G, Statistical analysis of the data presented in D and E (#P<0.05).

NAC is a thiol-containing compound that has been used as a promising antioxidant to counteract oxidative stress in many diseases. In the present study, we assessed the degree to which NAC supplementation was able to reduce or reverse the severity of CNV that formed under hyperglycaemic conditions. As shown in fig. 4, treatment with NAC significantly reduced the amount fluorescence leakage of CNV in diabetic mice (#*P*<0.05, Fig. 5 A). In addition, both the area (25899±5997 vs. 19616±5158 µm^2^ in STZ and NAC mice, respectively; #*P*<0.05; Fig. 5 B, C, and F) and width of the CNV lesions in NAC-treated mice (240.3±30.9 vs. 199.1±28.7 µm in STZ and NAC mice, respectively; #*P*<0.05; Fig. 5 D, E, and G) were decreased relative to the CNV characteristics in the control group. However, there was no significant difference in the thicknesses of the CNV lesions in the two groups (28.1±3.7 vs. 26.5±3.7 µmin STZ and NAC mice, respectively; *P*>0.05; Fig. 5 D, E, and G).

### NAC Supplementation Inhibited the Hyperglycaemia-induced Oxidative Stress and Expression of p-STAT3 and VEGF

**Figure 6:**
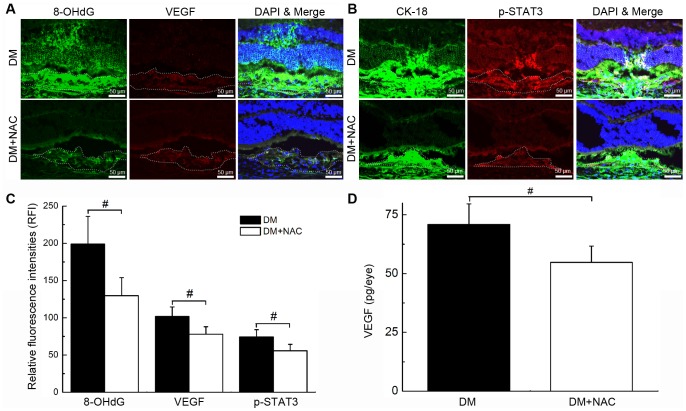
NAC supplementation inhibited hyperglycaemia-induced 8-OHdG, p-STAT3 and VEGF expression. A–B, Expression levels of 8-OHdG, VEGF and p-STAT3 in untreated diabetic (DM) and NAC-treated diabetic (NAC) mice. CNV lesions are indicated by dashed lines. C, Statistical analysis of the data in A and B (#P<0.05); D, Statistical analysis of the data from the VEGF ELISA (#P<0.05).

Consistent with our histology findings, NAC supplementation significantly reduced the severity of the hyperglycaemia-induced oxidative DNA damage (199.2±37.2 vs. 129.7±24.3 RFI in diabetic and NAC group, respectively; #*P*<0.05; Fig. 6 A and C), and expression of VEGF (101.8±12.8 vs. 78.1±9.9 RFI in diabetic and NAC group, respectively; #*P*<0.05; Fig. 6 A and C) and p-STAT3 protein (74.3±9.7 vs. 55.8±8.7 RFI in diabetic and NAC group, respectively; #*P*<0.05; Fig. 6 B and C). The NAC-induced decreased in the level of VEGF expression was further confirmed by the ELISA (70.9±8.8 vs. 54.8±6.9 pg/eye in diabetic and NAC group, respectively; #*P*<0.05; Fig. 6 D).

### Treatment with NAC and AG490 Inhibited p-STAT3 and VEGF Expression in RPE Cells that were Exposed to High Glucose Conditions

**Figure 7:**
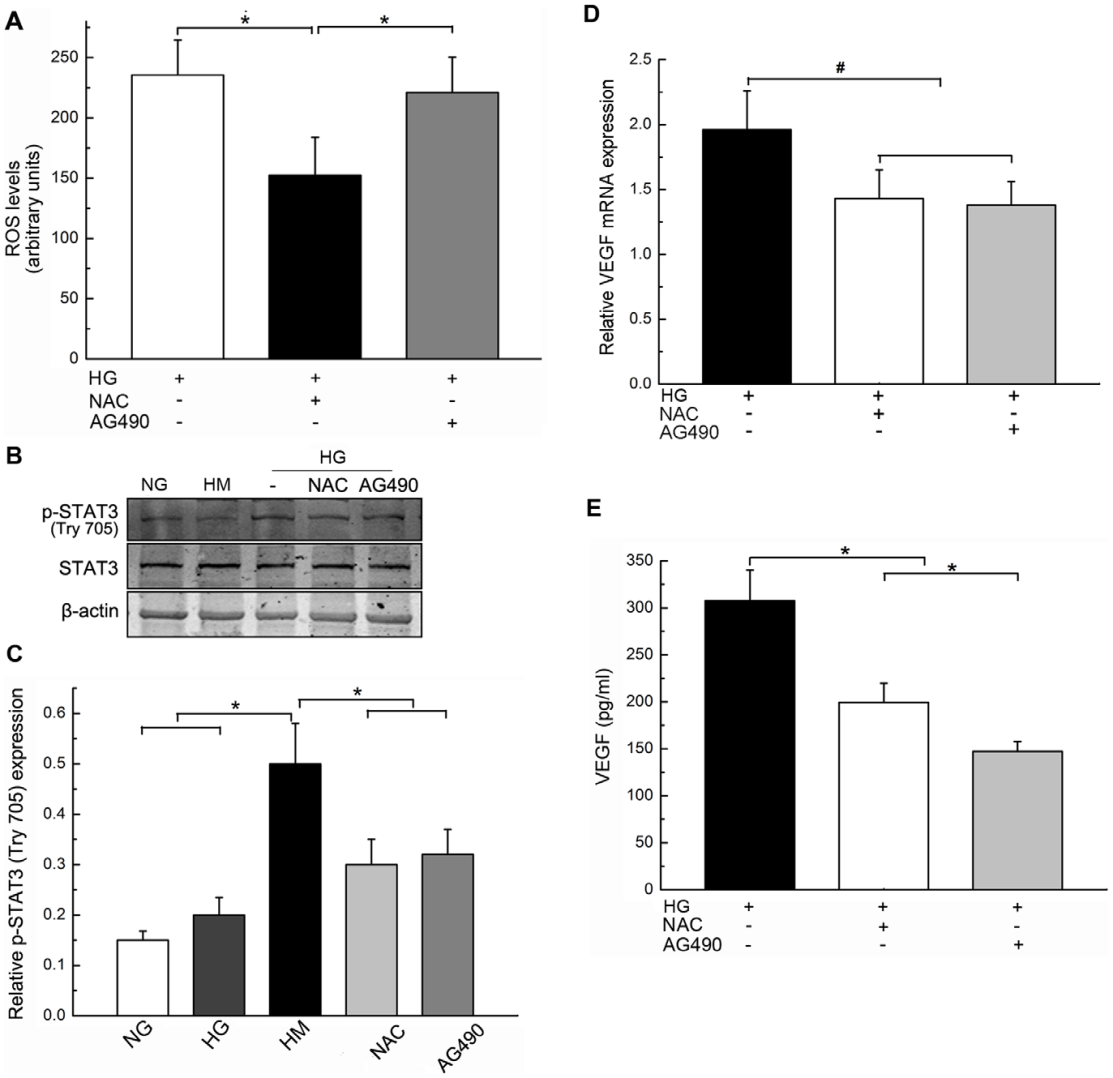
Treatment with NAC and AG490 inhibited p-STAT3 and VEGF expression in RPE cells that were exposed to high glucose (HG) conditions. RPE cells were cultured under normal glucose (NG) or high mannitol (HM) conditions and were subsequently treated with NAC or AG490 in the presence of an HG medium for 3 hours. A, Statistical analysis of the intracellular ROS data (*P<0.01). B, Representative data from Western blot analysis of p-STAT3 and total STAT3 expression in RPE cells. Data presented represent of three iterations of the experiments. C, Statistical analysis of the data in B (*P<0.01). D, Statistical analysis of the VEGF mRNA expression level data that were obtained by RT-PCR (*P<0.01). E, Statistical analysis of the VEGF ELISA data (*P<0.01, #P<0.05).

**Figure 8:**
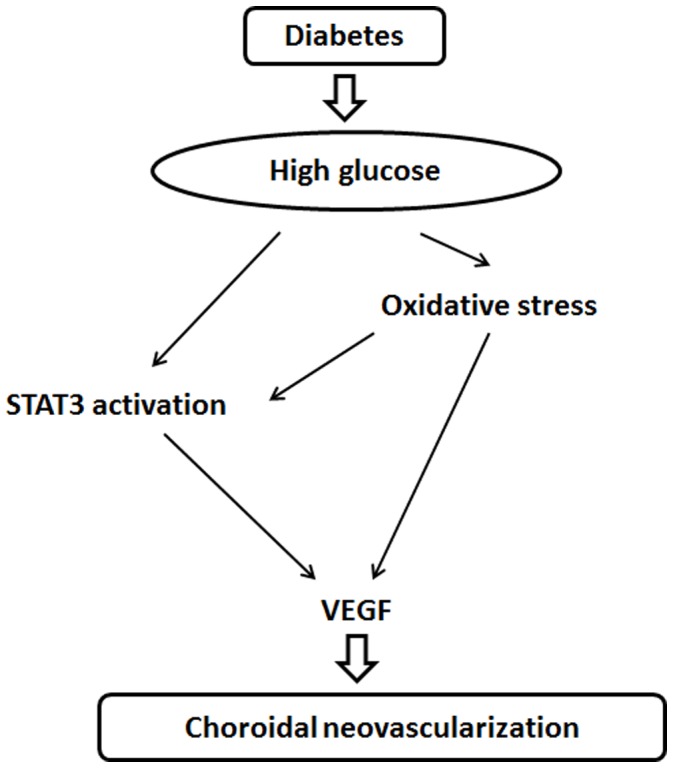
Mechanism for the diabetes-induced exacerbation of choroidal neovascularisation. Hyperglycaemia-increased oxidative stress is an upstream factor that promotes STAT3 activity, which in turn leads to the activation of VEGF transcription and eventually exacerbates the development of CNV.

To determine the relationship between oxidative stress and activation of the STAT3 signalling pathway, we tested the effects of NAC and the JAK2/STAT3 pathway inhibitor AG490 on RPE cells that were exposed to high glucose conditions. The administration of NAC reduced the degree of intracellular ROS formation; AG490 administration had no effect (*P<0.01, Fig. 7 A). The induction of p-STAT3 expression in RPE cells that were exposed to high glucose conditions was inhibited by both NAC and AG490 (*P<0.01, Fig. 7 B and C). RT-PCR and ELISA experiments confirmed that VEGF expression was inhibited by both NAC and AG490 (*P<0.01, #*P*<0.05, Fig. 7 D and E). Thus, our data suggest that oxidative stress can be considered an upstream factor that affects STAT3 activity, which in turn leads to the high glucose-mediated transcriptional activation of angiogenic genes such as VEGF (Fig. 8).

## Discussion

AMD is the primary cause of blindness among elderly people (those who are 65 years of age and older) in developed countries [26], [27]. There are two forms of AMD: “dry” AMD, which is characterised by the presence of soft drusen or geographic atrophy, and “wet” AMD, which is characterised by the presence of CNV under the macula. Approximately 10%–15% of dry AMD cases progress to the more advanced and damaging form of AMD, which is characterised by CNV that results in rapid and progressive central vision loss. It has been hypothesised that diabetes-related changes in the structures and functions of the RPE, Bruch’s membrane and the choroid layer result in an increased risk of developing AMD [9]. However, disagreement among current results from a number of epidemiological investigations and the limited number of mechanism-specific investigations mean the association between diabetes and AMD remains unclear. Retrospective study of Borrone R et al. found that the prevalence of ARM was lower in diabetic patients and even lower in patients with DR, but the exudative form (CNV) was higher than the atrophic form in diabetic patients compared to the general population [28]. Proctor B et al. have paid attention on the relationship between DR and ARMD [29]. They found that DR patients were much less likely to have CNV, but what’s the incidence of CNV in diabetic patients without DR is still unknown. While in the EUREYE study, a positive association of diabetes with CNV was found, but the atrophic form was not relevant with diabetes [7]. Taken together, diabetes with/without DR and different forms of AMD all suggest a different intraocular pathological environment for CNV development. Most of the epidemiological data focused on the relationship between AMD and diabetes remain controversial. Therefore, a lot more experimental researches focusing on the underlying mechanism needs to be further carried out.

**Table 1:** Numbers of animals per group and laser spots per eye that were included in each CNV assay.

Assay	Eyes	Laser spotsper eye	Statistical analysis method
FFA	7	6	Mann-Whitney U test
HE staining	3	6	Student’s *t* test
Choroidal flatmount	4	6	Student’s *t* test
Immunofluorescence	3	6	Student’s *t* test
ELISA	10	10	Student’s *t* test

In the current study, we observed that the CNV lesions in STZ-induced diabetic mice were significantly larger than similar lesions in wild-type mice, which indicated that diabetes might have an effect on the development of CNV. Diabetes is characterized by hyperglycaemia due to absolute or relative lack of insulin. Although animal model cannot fully mimic the clinical manifestations and pathological features, animal experiments have contributed much to our understanding of mechanisms of human disease. STZ can selective destruct β-cells of the pancreas, resulting in a lack of insulin secretion and finally increasing the blood glucose level. The model is stable and the effect is significant [30]. It has been reported that the duration of diabetes had no correlation with AMD, but the relationship needs to be further evaluated [1]. We induced CNV in a short period of time after STZ injection in order to observe the effect of hyperglycemia rather than the course of disease or chronic diabetic complications on CNV formation. Diabetes-induced hyperglycaemia has been implicated in the development of diabetes-specific pathology [31]. Histopathological studies of the eyes of diabetic patients have revealed thickening of the basement membranes in the walls of the choriocapillaris, luminal narrowing, dropout of the choriocapillaris, and thickening of Bruch’s membrane [32]. Findings from our study suggested that there is a positive relationship between diabetes-induced hyperglycaemia and the development of CNV. Glycaemic control is a very important aspect of diabetes management. Poor control of the blood sugar levels of diabetic patients can accelerate the progression of the disease and may increase the risk of diabetes-related complications [31], [33], [34]. This may help explain the controversial epidemiological results regarding the relation between diabetes and wet AMD. Recruitment biases and differences among study participants, measurement and statistical analyses may account for some of the variation among the results of these studies, but we noticed that most of them did not pay close attention to the control of blood sugar. Therefore, we confirmed for the first time that hyperglycaemia plays an important role in the exacerbation of CNV.

Previous studies have reported that the increased levels of VEGF and inter-cellular adhesion molecule 1 (ICAM-1) expression and the activation of a complementary system were associated with the development CNV in type 1 and type 2 diabetic rodent models [10], [11], but the underlying mechanism for these changes was still poorly understood. Hyperglycaemia-induced diabetic vascular damage has been identified as occurring via 4 major pathways: the activation of protein kinase C (PKC) isoforms; the formation of advanced glycation end-products; up-regulation of activity in the polyol pathway; and up-regulation of activity in the hexosamine pathway [35]. In all 4 of these mechanisms, oxidative stress has been considered a singular upstream event that is involved in promoting the process of pathology in diabetes and its related vascular complications [31], [36]. Moreover, oxidative stress has also been implicated in the up-regulation of VEGF expression and in pathological ocular angiogenesis [37], [38]. Thus, we examined the oxidative stress statuses of mice with experimentally induced CNV and the oxidative stress statuses of RPE cells that had been exposed to high-glucose environments. Increased levels of oxidatively modified DNA (8-OHdG), which is one of the most frequently used and reliable indicators of oxidative damage, were most frequently detected in experimentally induced CNV lesions in diabetic mice on the third day after laser injury. Meanwhile, elevated levels of ROS were also confirmed in RPE cells that had been exposed to high-glucose environments in vitro. Taken together, these findings imply that oxidative stress may contribute to the development of CNV in early stages of diabetes.

Because oxidative stress plays pivotal role in high-glucose-induced angiogenesis and CNV development, we sought to investigate whether antioxidant supplementation could hamper the development of CNV in hyperglycaemic conditions. NAC is a potent antioxidant that is known to be a precursor of glutathione (GSH). It has been reported that NAC acted directly as free radical scavengers and is independent of its ability to enhance GSH synthesis [39], [40]. A previous study discovered that NAC supplementation in a diabetic mouse model of an incisional wound resulted in lower levels of oxidative stress among the animals’ tissues [41]. It has also been reported that NAC administration prevented oxidative damage to RPE cells that was caused by exposure to a cigarette smoke extract that induced oxidative injury and contributed to the progression of AMD [42]. Our results demonstrated that NAC administration effectively alleviated oxidative stress levels that were exposed to hyperglycaemic conditions in mice and in cultured RPE cells. In addition, we further determined that NAC treatment was able to diminish the severity of CNV in diabetic mice.

RPE cells have the ability to respond quickly and adaptively to environmental stressors by expressing a number of genes that promote the development of CNV. Signalling pathways that regulate the biological functions of RPE cells are useful for understanding the molecular mechanisms that underlie the development of CNV [43]. STAT3 is a cytoplasmic transcription factor that transmits extracellular signals to the nucleus; activated STAT3 in the nucleus then binds to specific DNA promoter sequences and regulates gene expression [44]. In early stages of experimentally induced CNV in diabetic mice and in RPE cells when exposed to hyperglycaemic enviroments, we provided the first evidence that the level of p-STAT3 was dramatically up-regulated and accompanied by increased oxidative stress and upregulation of VEGF. While treatment with NAC was found to suppressed the level of p-STAT3 and VEGF overexpression in vivo and in vitro. After exposing to a JAK2/STAT3 pathway inhibitor AG490, STAT3 activation was blocked, which ultimately lead to a decrease in the intracellular level of VEGF mRNA and protein expression in RPE cells, suggesting that the activation of the STAT3 signalling pathway triggers VEGF expression. However, we found that AG490 had no effect on the intracellular level of ROS. Thus, our results indicated that STAT3 signalling may activated by ROS in RPE cells and participate in the development of CNV under hyperglycaemic conditions.

In summary, we confirm for the first time that hyperglycaemia plays a pivotal role in the diabetes-aggravated development of CNV in mice. The underlying mechanism might involve an increase in the level of oxidative stress that results in CNV and the subsequent activation of STAT3-regulated VEGF expression in RPE cells. Furthermore, our data provide evidence that treatment with NAC effectively rescues the severity of experimentally induced CNV in diabetic mice. Our findings suggest that diabetes is a risk factor for disorders that involve the development of CNV, and antioxidant treatment may represent a therapeutic strategy for treating these diseases.

## Materials and Methods

### Ethics Statement

The experimental protocol that was used in the present study was approved by the Institutional Care and Use Committee at the Fourth Military Medical University (FMMU). All of the mouse studies were approved by the Animal Studies Committee at FMMU.

A total of 108 wild-type C57BL/6J mice, all of which were 8-week-old males and were obtained from the experimental animal centre at FMMU, were used in the present study. The mice were randomly divided into three groups: a control group (n = 27), a diabetic group(n = 54), and a diabetic group that was treated with N-acetyl-cysteine (NAC, n = 27). STZ-induced diabetic mice received daily intraperitoneal injections of the anti-oxidant NAC (Sigma Chemical, St. Louis, MO, 200 mg/kg/day) after laser photocoagulation. After 2 weeks of monitoring diabetes in the diabetic mice, laser induction of CNV was administered to the right eyes of all three groups of mice.

### Generation of Diabetic Mice

To induce diabetes in our model animals, mice received daily intraperitoneal injections of STZ (Sigma Chemical, St. Louis, MO, 60 mg/kg of body weight in 0.05 M sodium citrate buffer at a pH of 4.5) for 5 days. Control animals were injected with citrate buffer only. Seven days after the fifth injection, the blood glucose levels of the animals were measured using a glucomonitor. Tail vein blood was used for blood glucose analyses. Mice with glucose levelsabove 300 mg/dl were considered as hyperglyceamic [45].

### CNV Induction in Mice

A laser procedure was used to induce CNV in the right eyes of the mice in this study, and the induction of CNV was performed according to a previously reported protocol [46]. Briefly, the mice were anesthetised, and their pupils were dilated. Laser photocoagulation with a wavelength of 532 nm, a spot size of 75 µm, a duration of 0.1 seconds, and an intensity of 90 mW was delivered using a slit lamp and a corneal contact lens. The burns were performed at positions that were 1.5–2 disc diameters away from the optic nerve. Only those laser spots at which the rupture of Bruch’s membrane was confirmed via the presence of a vaporisation bubble and the absence of haemorrhaging were considered successful and were included in the study.

### CNV Severity Evaluation

CNV (6 spots per eye) was evaluated using fundus fluorescence angiography (FFA) 2 weeks after the photocoagulation procedure. The mice were anesthetised and were given intraperitoneal injections that contained 0.1 ml of 2.5% sodium fluorescein (Wuzhou Pharmaceutical, Guangxi, China). FFA recording began 3 min after the injection, and recordings were performed with a digital imaging system (Heidelberg Engineering, Heidelberg, Germany). The presence of a hyperfluorescent lesion at a site of laser irradiation that increased in size was defined as leakage and was indicative of both the incidence of CNV and the leaking grade of it. Two examiners graded fluorescence leakage independently using reference angiograms. Fluorescein leakage intensity scores were graded as follows: 0 = no leakage; 1 = slight leakage; 2 = moderate leakage; 3 = prominent leakage.

Choroidal flat mounts were prepared on day 14 after CNV induction (6 spots per eye) in accordance with a previously described protocol [47]. Anesthetised mice were transcardially perfused with a 0.9% saline solution followed by a 4% paraformaldehyde solution. The entire ocular globes were enucleated, and the anterior segment and neural retina were removed from each globe. The remaining RPE-choroid-sclera complex was flatmounted using six or more radial cuts, after which the flatmount preparations were permeabilised in a 0.2% Triton X-100 solution for a period of 24 h prior to transferring them to a 1∶1000 solution of rhodamine-conjugated Ricinus communis agglutinin (Vector Laboratories, Burlingame, CA, USA). Choroidal preparations were incubated with the agglutinin for 24 h and were subsequently washed in a 0.01 M Tris-Buffered Saline Tween-20 (TBST) solution for another 24 h. Flatmounts were subsequently examined and photographed using confocal laser scanning microscopy (Olympus Corporation, Tokyo, Japan), and the CNV area of each preparation was assessed using the image pro plus software program (IPP 6.0). Individual lesions with surface areas of more than 0.50 disc areas (DAs) were defined as having CNV.

Histopathological analysis was performed according to a previously described procedure [48]. Mice that were examined using light microscopy were killed on the 14th day after photocoagulation (6 spots per eye), and their eyes were enucleated. Eyecup preparations were fixed via incubation in Bouin’s fixative (Zhongshan Biotechnology Company, Beijing, China) at 4°C for a period of 24 h. The fixed tissues were embedded in paraffin, serially sectioned into 3-µm slices, and stained with hematoxylin and eosin. Serial slices were examined, and the specimens that contained the thickest and/or widest lesions among the set of specimens that was obtained for each instance of CNV were evaluated. Sections that had been stained with hematoxylin and eosin were digitised using a light microscope (Olympus Corporation, Tokyo, Japan) that was connected to a colour video camera equipped with a frame grabber. IPP 6.0 was used to calculate the maximum thicknesses and lengths of each CNV from the selected hematoxylin and eosin-stained specimens.

### 8-OHdG, p-STAT3 and VEGF Immunofluorescence

On day 3 after photocoagulation (6 spots per eye), anesthetised mice were transcardially perfused with a 0.9% saline solution followed by a 4% paraformaldehyde solution. Eyes were then enucleated and post-fixed. Alternate sets of serial vertical sections of the eye were cut and mounted. To detect the level of oxidative DNA damage and the expression of p-STAT3 and VEGF protein in or near sites at which CNV occurred, serial cross sections of the eyes were incubated with primary antibodies against 8-OHdG (1∶200, Biosynthesis Biotechnology Company, Beijing, China), p-STAT3 (1∶200, Santa Cruz Biotechnology, CA, USA), VEGF (1∶200, Santa Cruz Biotechnology, CA, USA) and CK18, which is an RPE marker (1∶100, Biosynthesis Biotechnology Company, Beijing, China). Prepared sections were then incubated with FITC and TRITC-conjugated secondary antibodies, counterstained with DAPI, and examined with confocal laser scanning microscope using identical intensity of laser stimulation. IPP 6.0 was used to assess the relative fluorescence intensities (RFI) by dividing the average luminosity within the lesion by the average luminosity of the nomal choroid away from the CNV.

### Cell Culture

Human RPE cells were obtained from a mature cell line that had been preserved in our laboratory as has been previously described [43]. Experimentation was carried out on subconfluent RPE cells in passage numbers three through eight. Cells in the control group were cultured and maintained in Dulbecco’s Modified Eagle Medium (DMEM) with a normal glucose concentration (NG, 5.5 mM D-glucose) that was supplemented with 10% newborn serum in a humidified 5% CO_2_ incubator at 37°C. Cultured RPE cells in other experimental groups were treated with a high mannitol control medium (HM, 5.5 mM D-glucose+24.5 mM D-mannitol), a high glucose medium (HG, 30 mM D-glucose), an antioxidant (1 mM NAC with HG), or a Janus kinase (JAK)-specific inhibitor (30 µM AG490 with HG).

### ELISA for VEGF

The ocular levels of VEGF protein expression on day 3 after photocoagulation (10 spots per eye) were determined using a mouse VEGF ELISA kit (USCN Life Science and Technology, Wuhan, China). On the third day after photocoagulation, the eyes were removed and prepared for ELISA according to a previously reported protocol [46]. The eyes were quick-frozen in 200 µl of phosphate-buffered saline solution (pH 7.4) that contained 0.05% phenylmethylsulfonyl fluoride, and they were then manually homogenised on ice and exposed to three freeze-thaw cycles in liquid nitrogen and wet ice. The homogenates were centrifuged in a refrigerated desktop centrifuge to pellet any insoluble material, and the supernatants were collected. ELISA was performed according to the instructions from the manufacturer.

A human ELISA kit was used to measure the expression levels of VEGF protein that were secreted by human RPE cells in various culture media at the appropriate times (USCN Life Science and Technology, Wuhan, China) in accordance with the manufacturer’s instructions.

Representative results were taken from three independent experiments and were expressed in picograms/millilitre.

### Measurement of Intracellular Oxidative Stress by Flow Cytometry

Analysis of high glucose-induced generation of intracellular oxidative stress was determined by flow cytometry using the 2′,7′-dichlorofluorescein diacetate (DCFH-DA) probe (Beyotime Institute of Biotechnology, Shanghai, China). DCFH-DA is a oxidation-sensitive nonfluorescent precursor dye that can be oxidized by H_2_O_2_, other ROS and low molecule weight peroxides to fluorescent DCFH. DCFH is generally considered a probe not only for H_2_O_2_ in presence of cellular peroxidases but also for the determination of ONOO•, and HO•. When used in cellular systems, DCFH is a general marker of oxidative stress rather than a specific indicator H_2_O_2_ formation or other ROS and reactive nitrogen species [49].

RPE cells were seeded on 6-well plates at 1×10^5^ cells per well and cultured for 24 h. After maintained in DMEM with different glucose concentration and various agents, cells were detached by means of trypsinisation, and a FAC Scan flow cytometer (BD, San Jose, CA) was used to measure the intensity of the cellular fluorescence intensity after a 30-min incubation in a 10 µmol/l DCFH-DA solution. The excitation and emission wavelengths were set at 488 nm and 525 nm respectively. Three independent experiments were performed and the results were expressed as the means ± SDs in arbitrary units of DCFH fluorescence intensity.

### RNA Preparation and Semi-quantitative RT-PCR Analysis

Trizol (Takara, Kyoto, Japan) was used to extract the total cellular RNA from RPE cells, and the extracted RNA was subsequently quantified. Two micrograms of total RNA was reverse-transcribed to cDNA using the Exscript RT Reagent kit (Takara, Kyoto, Japan). All of the PCR experiments were performed using Taq polymerase (Promega, Madison, WI, USA); the primers 5′-AGGAGGGCAGAATCATCACG-3′ and 5′-CAAGGCCCACAGGGATTTTCT-3′ were used in PCR for VEGF, and 5′-GCCTCAAGATCATCAGCAAT-3′ and 5′-AGGTCCACCACTGACACGTT-3′ were used for the GAPDH control. Agarose gels that had been stained with ethidium bromide (1%) were scanned using a Fluor-Multimager (BioRad). IPP 6.0 was used to quantitate the band intensities of the PCR products. All experiments were repeated at least three times.

### Western Blot Analysis

RPE cells were collected and lysed, and the expression levels of various proteins in the RPE cells were measured. Standard techniques were used to assess the expression levels of p-STAT3, STAT3 and β-actin. The following primary antibodies were used: monoclonal mouse anti-STAT3 (1∶200, Santa Cruz Biotechnology, USA), monoclonal mouse anti-p-STAT3 (1∶200, Santa Cruz Biotechnology, USA) and polyclonal rabbit anti-β-actin (1∶100, Santa Cruz Biotechnology, USA). All experiments were repeated at least three times.

### Statistical Analysis

Statistical analyses were performed using the SPSS 13.0 software program. Data from several experiments were pooled and subsequently presented as the means and standard deviations. One-way analyses of variance (ANOVAs) followed by LSD-t tests were used to make comparisons between pairs of groups. Student’s *t* tests were used for the remaining statistical analyses. All of the experimental datasets were scrutinised to ensure that the sample variance was normally distributed, and appropriate non-parametric tests were applied when necessary. A two-tailed p-value of *P*<0.05 was considered significant.

The numbers of animals in each group and laser spots per eye have been summarised in Table 1; the statistical analyses that were used in each assay are also summarised in Table 1.
